# Career-Specific Parenting Practices and Career Decision-Making Self-Efficacy Among Chinese Adolescents: The Interactive Effects of Parenting Practices and the Mediating Role of Autonomy

**DOI:** 10.3389/fpsyg.2019.00363

**Published:** 2019-02-21

**Authors:** Yu Chi Zhang, Nan Zhou, Hongjian Cao, Yue Liang, Shulin Yu, Jian Li, Linyuan Deng, Ruixi Sun, Qinglu Wu, Ping Li, Qing Xiong, Ruihong Nie, Xiaoyi Fang

**Affiliations:** ^1^Institute of Developmental Psychology, Beijing Normal University, Beijing, China; ^2^Faculty of Education, Beijing Normal University, Beijing, China; ^3^Department of Psychology, School of Education, Guangzhou University, Guangzhou, China; ^4^Faculty of Education, University of Macau, Macau, China; ^5^Department of Social Work, The Chinese University of Hong Kong, Shatin, China

**Keywords:** career decision-making self-efficacy, career-specific parenting practices, Chinese adolescents, autonomy, high school

## Abstract

This study examined the unique and interactive effects of various career-specific parenting practices (i.e., parental career support, interference, and lack of engagement) on Chinese high school students’ career decision-making self-efficacy (CDSE) as well as the mediating role of autonomy in such associations. Based on data from 641 Chinese high school students (47.6% male; mean age = 15.28 years old, *SD* = 0.49) in 2016, two moderated mediating effects were identified. Higher level of parental career engagement strengthened the positive association between parental career support and adolescents’ autonomy, which in turn, was associated positively with adolescents’ CDSE. Parental career interference related negatively with adolescents’ CDSE via autonomy when lack of parental career engagement was low, but related positively with adolescents’ CDSE via autonomy when lack of parental career engagement was high. These findings advance our understanding of the underlying processes between career-specific parenting practices and adolescents’ CDSE. Implications for practices were discussed.

## Introduction

Career decision-making self-efficacy (CDSE), which refers to one’s *confidence in one’s ability to engage in educational and occupational planning and decision making*, is a critical indicator of adolescent career competence (Luzzo, 1993; Chiesa et al., 2016). CDSE typically serves as an important trigger to promote career outcomes, including career decision-making skills (Luzzo, 1993; Choi et al., 2012), career planning outcome expectations (Gushue, 2006), stable career identity (Cordeiro et al., 2015), and lower career indecision (Guay et al., 2003). CDSE is especially critical during high school years when adolescents are supposed to make important career-related decisions. Students who are highly confident about their capacity in making better career decisions would have more interest to nurture their career goals, devote more time to their career explorations, and may probably make better career decisions ultimately (Gushue et al., 2006; Chiesa et al., 2016). Thus, it seems warranted to identify the antecedents of adolescents’ CDSE (Miles and Naidoo, 2017).

Research reveals that parenting practices have been implicated in the development of adolescents’ CDSE (Gushue and Whitson, 2006; Sovet and Metz, 2014). However, previous research is limited in several ways. First, most of the research on parenting and CDSE focuses on the effects of general parenting practices (e.g., parental support) with few studies examining associations between career-specific parental practices in relation to adolescents’ CDSE (Garcia et al., 2012). In fact, career-specific parental practices may yield stronger relations with adolescents’ career development than general parenting practices (Tracey et al., 2006). Second, the slim body of research that examined career-specific parenting practices and CDSE typically examined a given type of career-specific parental practices (e.g., parental career support) (Keller and Whiston, 2008). Different career-specific parental practices, however, may have unique or interactive effects on career development (Guan et al., 2015). Thus, it is unclear which type of career-specific parental practices would play a more vital role in shaping adolescents’ CDSE, and whether different career-specific parental practices cooperate with each other in relation to adolescents’ CDSE.

Furthermore, the underlying mechanisms linking career-specific parenting practices to adolescents CDSE are not well understood. Self-determination theory (SDT) appears to provide a conceptual framework to explaining this underlying process (Deci and Ryan, 1985, 2000). SDT suggests that individual’s development is promoted and sustained by individual-context interaction process. As one of the three important psychological needs, autonomy may serve as the origin and motivating agent of adolescents’ development. Social contextual factors (e.g., parental practices) could promote or hinder adolescents’ developmental outcomes through facilitating or forestalling the development of autonomy. Thus, autonomy may serve as a linking mechanism in the associations between career-specific parenting practices and CDSE. Taken collectively, the goal of this study is to examine the direct and interactive effects of test career-specific parenting practices on Chinese high school students’ CDSE as well as the mediating role of autonomy in such associations.

### Career-Specific Parenting Practices and Adolescents’ CDSE

Bandura (2002) proposed that self-efficacy was domain-specific and CDSE is the key indicator of self-efficacy in the face of career decision (Choi et al., 2012). Social cognitive career theory (SCCT) posits that parenting practices could engender and increase individuals’ career self-efficacy via several key self-efficacy learning experiences, including performance accomplishments, vicarious learning, social persuasion, and emotional arousal (Taylor and Betz, 1983; Lent et al., 1994). During the transition to adulthood, adolescents encounter many new options and uncertainties, and thus parental career support could be critical sources of guidance whereas parents’ non-participative, neglectful or intrusive parenting practices may hinder adolescents’ career planning, explorations, and the development of stable ego identity (Mortimer et al., 2002; Lim and Loo, 2003; Pellerone et al., 2017a,b). Specifically, parental career support (e.g., expressions of interests and concerns, encouragement, instrumental assistance, and emotional backing) could provide adolescents with positive career feedback (i.e., social persuasions) and thus may promote adolescents’ confidence to embrace career challenges and to make career decisions (Zhao et al., 2012; Guan et al., 2016). In contrast, both parent career interference (i.e., parents’ imposing their personal ideas on children’s career directions and choices) or lack of parental career engagement (i.e., parents’ inability or reluctance to get involved in their children’s career development) may convey to adolescents that he or she is not competent and not capable to make career decision (Lim and Loo, 2003). In support of the hypotheses, Zhao et al. (2012) sampled 196 Singaporean university students and found that parental career support was related positively to youth’s career self-efficacy and the lack of parental career engagement was related negatively to career self-efficacy.

In addition to the main associations between career-specific parenting practices and adolescents’ CDSE, these parenting practices may also be configured in different patterns within individuals such that parental career support, interference, and lack of engagement may interplay with each other in shaping adolescents’ career developmental outcomes (Dietrich and Kracke, 2009; Guan et al., 2015). Dietrich and Kracke (2009) found in a sample of 359 German adolescents that adolescents’ perceived parental career support interacted with either career interference or lack of engagement in the prediction of adolescents’ career decision-making difficulties. Specifically, the positive association between parental career support and adolescents’ career exploration was stronger for adolescents who perceived higher parental career interference or those who perceived higher levels of parental lack of engagement. Guan et al. (2015) analyzed data from 244 Chinese undergraduates and their parents and found a similar interaction pattern involving parental career support and lack of engagement in the prediction of students’ career exploration. Moreover, they also found that the negative effects of interference on career exploration were stronger among students with lower level of lack of parental career engagement.

Overall, the current literature on career-specific parenting are limited in several ways. First, most of the empirical studies on parenting practices and career development almost exclusively focused on general parenting practices or just parental career support (Lim and Loo, 2003; Tracey et al., 2006; Keller and Whiston, 2008; Garcia et al., 2012; Guan et al., 2016; Pedro et al., 2016). Thus, little is known about the differential associations between various aspects of career-specific parenting practices (i.e., parental career support, interference, and lack of engagement uniquely) and adolescents’ career developmental outcomes, not to mention CDSE. Second, the interactions among career-specific parenting in relation to CDSE is not clear yet. To address these gaps, the first aim of this study was to examine the differential (i.e., unique) and the interactive effects of various career-specific parenting practices on adolescents’ CDSE.

### The Mediating Role of Autonomy

Most of the research focused on the direct associations between parenting practices and adolescents’ career development. Thus, little is known about the underlying mechanisms that may explain such associations (Guay et al., 2003; Pesch et al., 2016). From a SDT perspective, autonomy may be a highly potential mediator (Deci and Ryan, 1985, 2000). Autonomy refers to the need to feel self-volition (Grolnick et al., 1991; Deci and Ryan, 2000). As one of three critical psychological needs, autonomy is considered as a crucial developmental task during adolescence and the developmental process may be impelled by continuous interactions between autonomy and dispositional integrative and social environmental influences (e.g., parenting practices; Deci and Ryan, 2000; Friedman et al., 2009).

First, parental support behaviors may facilitate adolescents’ autonomy via encouragement of career planning and providing suggestions and directions for career explorations whereas both parental interference and lack of engagement may limit adolescents’ opportunities to actualize self-governed career explorations and thus impede the development of autonomy. Second, autonomy may play a substantial role in promoting adolescents’ CDSE via motivating adolescents to establish their career goals and to explore career interests (Deci and Ryan, 1985; Grolnick et al., 1991; Guay et al., 2003; Friedman et al., 2009; Zhou et al., 2009; Doren and Kang, 2016). Thus, career-specific parenting practices may be closely related to the need fulfillment of adolescents’ autonomy in their career planning, explorations, and actions, which ultimately may lead to enhanced CDSE.

Several seminal work has supported the associations between career-specific parenting practices and adolescents’ autonomy. Dietrich and Salmela-Aro (2013) conducted a longitudinal study on 807 Finnish adolescents and found that parents’ career support was associated positively with, and lack of parental uniquely related negatively to adolescents’ autonomous motivation 3 years later. Moreover, Costa et al. (2016) found that highly controlled parenting behaviors disrupted the adolescents’ autonomy and led to heightened depression. This finding provide evidence that parental career interference would hinder the development of autonomy by limiting adolescent’s opportunity to engage in self-governed career explorations and established their own career goals. Although the SDT and empirical studies have highlighted the importance of autonomy in shaping adolescents’ developmental outcomes (Deci and Ryan, 2000; Pesch et al., 2016), to our knowledge, only one study explicitly examined the relation between autonomy and CDSE (Guay et al., 2003). Guay et al. (2003) sampled 834 college students and found a positive association between career decision autonomy and CDSE. This finding suggests that adolescents with higher autonomy may possess higher intrinsic motivation to set up career goals, to explore career interests and practice problem solving skills, which inevitably would facilitate their CDSE.

### The Cultural Context

Previous research on relations between parenting practices and adolescents’ CDSE has been primarily conducted in the Western culture (Keller and Whiston, 2008) and thus investigations in the Chinese context may be important because of the following considerations. First, the unique socioecological niches in which adolescents inhabit affect the importance of parenting practices in their children’s career development. Although Chinese adolescents still need to navigate through the critical transition after completion of high school, the heightened pressure from Chinese college entrance examination prevent them from devoting sufficient time into establishment of career goals, making career plans, exploring career options (Fleisher, 2014). Without official national curriculum or programs to guide high school students’ career development in China, Chinese parents’ career-specific parenting behaviors are supposed to be of more importance to their adolescent children’s career development as compared to their Western counterparts.

Second, in the more collectivistic cultural context (e.g., China), adolescents are supposed to consider significant others’ opinions and family obligations when making career decisions (Pan et al., 2013; Sovet and Metz, 2014). Moreover, consistent with the traditional cultural filial piety, adolescents are instructed to endorse parental power and control rather than autonomy (Chirkov, 2009; Pan et al., 2013). Nonetheless, fulfillment of autonomy is a cross-cultural universal psychological need (Deci and Ryan, 1985; Zhou et al., 2009) and thus it is not clear autonomy could serve as a linking mechanism in the association between career-specific parenting practices and Chinese adolescents’ CDSE. Taken together, examinations of associations among career-specific parenting practices, autonomy, and Chinese adolescents’ CDSE may provide nuanced understanding of the importance of career-specific parenting practices in adolescents’ career development and the applicability of SDT in the non-western culture, and also may provide important implications for Chinese adolescents’ career guidance practices (Chirkov, 2009; Pan et al., 2013).

### The Present Study

Based on data from 641 Chinese high school students, the present study examined: (1) the unique associations between three career-specific parenting practices (i.e., parental career support, interference, and lack of engagement) and Chinese high school students’ CDSE; (b) the interactive effects of the three career-specific parenting practices on Chinese high school students’ CDSE; and (c) the mediating role of autonomy in the associations between direct and interactive effects of the three career-specific parenting practices on Chinese high school students’ CDSE. Child gender and parents’ educational levels were specified as controls given their potential associations with parental career parenting practices and adolescents’ CDSE (Gianakos, 2001; Guay et al., 2003; Patel et al., 2008; Choi et al., 2012; Hsieh and Huang, 2014).

## Materials and Methods

### Participants and Procedures

**FIGURE 1:**
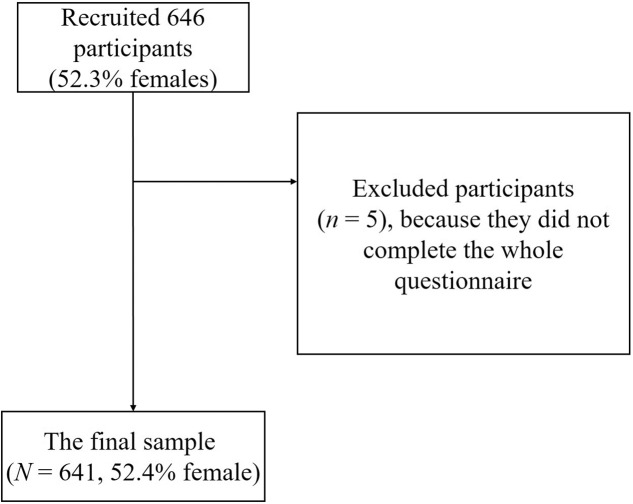
The consort flow diagram of participant recruitment.

This study was meant to examine family processes and adolescents’ career development. Participants were recruited through contacts with a public school in Beijing, China. Informed consent forms were taken home by students and completed by both parents and adolescents. All the parents permitted adolescents and all the adolescents themselves agreed to participate in the study, resulting in a sample of 646 participants. Five participants (three females and two males) were excluded because they did not complete the full questionnaires. The final sample comprised 641 (52.4% female) 10th graders (response rate = 99.40%) (Figure 1). Students’ age ranged from 14 to 16 years old (*M* = 15.28, *SD* = 0.49). Students’ expected highest educational achievement ranged from junior college education to doctoral degrees with 47.90% of them anticipating a doctoral degree in future. Students’ family socioeconomic status (SES) in the present study was indicated by parents’ highest education level. Specifically, 82.7% of students’ fathers and 74% of their mothers had at least college education.

The participating students completed the questionnaires in their classrooms. A research assistant explained to students about the purpose of the study. Students were informed that the information collected was totally anonymous and would only be used for research. The survey lasted for about 30 min. Every participant get a gift (about $1) after they completed the questionnaires. The project was approved by the university’s institutional review board (IRB).

### Measures

#### Career-Specific Parenting Practices

Career-specific parenting practices were assessed using the 15-item career-specific parental behaviors scale (Dietrich and Kracke, 2009). Participants rated the items on a 5-point Likert scale ranging from 1 (*does not apply at all*) to 5 (*applies perfectly*). This scale contains three subscales: parental career support (e.g., “*My parents encourage me to seek information about vocations I am interested in*.”), career interference (e.g., “*My parents would talk me out of a vocation they don’t like*.”), and lack of career engagement (e.g., “*My parents don’t care about my vocational preparation*.”). Mean scores were computed and used in the analyses and higher scores indicate that participants perceived their parents were more likely to employ particular behaviors. Previous studies have demonstrated that the PCB has good reliability and validity in Chinese samples (e.g., Guan et al., 2015). In the present study, Cronbach’s alphas were 0.90 for parent career support, 0.88 for parent career interference, and 89 for lack of parent career engagement.

#### Autonomy

Autonomy was assessed using the 20-item Worthington Autonomy Scale (WAS) (Anderson et al., 1994). This scale includes two subscales: the behavioral autonomy (e.g., “*I apologize for my part of an argument even if the other person doesn’t*.”) and the emotional autonomy (e.g., “*I can be close to someone and give them space at the same time*.”) subscales (Hmel and Pincus, 2002). Each subscale consists of 10 items. Participants responded on a 4-point scale ranging from 1 = *strongly disagree* to 4 = *strongly agree*. Mean scores were computed and used in the analyses and higher scores indicate greater autonomy. It has demonstrated good validity and reliability in Chinese samples (Zhou et al., 2009). In the present study, Cronbach’s alphas were 0.72 for behavioral autonomy and 0.74 for emotional autonomy.

#### Career Decision-Making Self-Efficacy (CDSE)

Career decision-making self-efficacy were measured using the 25-item Career Decision-Making Self-efficacy Scale-Short Form (CDMSE-SF) (Betz et al., 1996). The scale asked participants to indicate their agreement with statements on a 5-point Likert scale ranging from 1 = *no confidence at all* to 5 = *complete confidence* (e.g., “*I am able to make a plan of my goals for the next 5 years*.”). Mean scores were calculated and used in analyses and higher scores represent higher CDSE. Previous studies based on Chinese samples have demonstrated good reliability and validity of CDSE (Hampton, 2006; Garcia et al., 2015). Cronbach’s alpha was 0.94 in the present study.

#### Covariates

Students reported their gender (1 = *female*, 0 = *male*) and their fathers’ and mothers’ educational levels (1 = *primary school or less*, 2 = *junior high school*, 3 = *senior high school*, 4 = *vocational school*, 5 = *college education*, 6 = *master education*, 7 = *doctoral education*).

### Data Analysis

Hypotheses were tested using Structural Equation Modeling in Mplus 7.4, and missing values were handled using full information maximum likelihood estimation method (FIML; Acock, 2005). Parameters were estimated using maximum likelihood estimation. Although a non-significant chi-square statistic indicates a good model fit, a significant chi-square was expected for most models because of the large sample size. Therefore, other fit indices also were examined. Good model fit was indicated by the comparative fit index (CFI) values greater than 0.95, the root mean square error of approximation (RMSEA) values less than 0.05, and the standardized root mean square residual (SRMR) values less than 0.05 (Browne and Cudeck, 1993; Hu and Bentler, 1999; Byrne, 2001).

**FIGURE 2:**
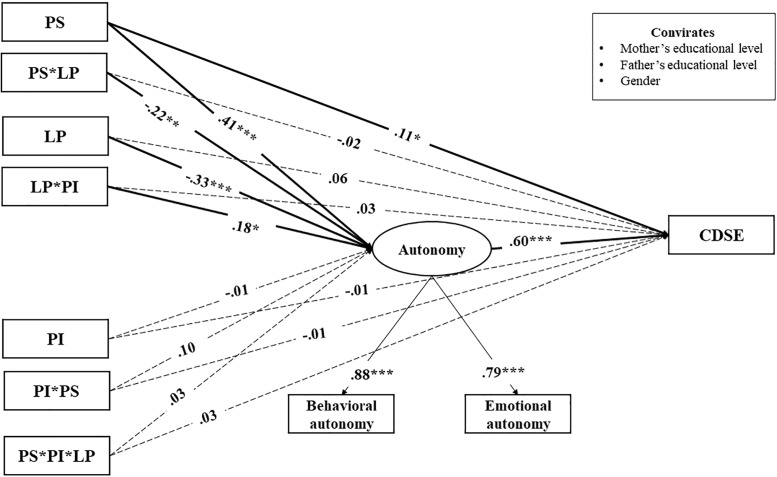
Associations among career-related parenting practices, autonomy, and CDSE. PS, parental career support; PI, parental career interference; LP, lack of parental career engagement; CDSE, career decision-making self-efficacy. To simplify presentation, the correlations between independent variables, and the correlation lines and predicting pathways involving covariates are not shown in the figure. Values are standardized coefficients. Solid lines indicate relations that were significant at *p* < 0.05. Parameter estimates for pathways that were not statistically significant at *p* < 0.05 (two-tailed) are depicted in dash lines in the figure. ^∗^*p* < 0.05, ^∗∗^*p* < 0.01, ^∗∗∗^*p* < 0.001 (two-tailed).

To test the direct and interactive effects of various career-specific parenting practices on adolescents’ CDSE and the mediating role of autonomy, we specified a moderated-mediation model (Figure 2). Specifically, parental career support, interference, and lack of engagement as well as the three-way and two-way interactions among and between these three parenting practices were specified as exogenous variables; autonomy was included as the mediating variable; and adolescents’ CDSE was specified as the endogenous variable. Youth gender and parental education were included as control variables. The three career-specific parenting practices were correlated to account for their relatedness. Indirect effects were evaluated using bootstrapping, a non-parametric, resampling strategy to calculate indirect effects with no assumption about the shape of sampling distribution of the coefficients (Preacher et al., 2007). Specifically, for a given 95% bootstrapped confidence interval, if zero is not included in the confidence intervals, the mediating effects are different from zero with 95% confidence (Hayes, 2009).

## Results

Descriptive statistics and correlations for the key study variables are presented in Table 1. Correlations among parental career support, interference, lack of engagement, emotional autonomy, behavioral autonomy, and adolescents’ CDSE were significant and in the expected directions. Given that all the data were collected at the same time using adolescents’ self-reports, we used Harmon’s one-factor test to examine potential common method bias (Podsakoff and Organ, 1986). We performed an exploratory factor analysis of all the items in this study. The first unrotated factor accounted for 38% of the total variance (less than 50%), indicating that the common method variance in the present study was minimum (Podsakoff and Organ, 1986).

**Table 1:** Descriptive statistics and correlations (*N* = 641).

	1	2	3	4	5	6	7	8
1 Gender (male = 1)								
2 Father’s educational level	-0.001							
3 Mother’s educational level	0.04	**0.62**						
4 Parental career support	0.05	**0.15**	**0.15**					
5 Parental career interference	-0.004	0.03	-0.06	**-0.15**				
6 Lack of parental career engagement	-0.07	-0.13	**-0.15**	**-0.44**	**0.43**			
7 Autonomy	0.07	0.07	0.06	**0.45**	**-0.16**	**-0.36**		
8 CDSE	-0.03	0.09	**0.09**	**0.40**	**-0.09**	**-0.21**	**0.57**	
*M*	47.6	4.18	3.99	4.26	2.22	1.71	3.31	3.89
*SD*	–	0.99	0.98	0.74	0.96	0.77	0.34	0.59


*Bold correlations are significant at p < 0.01. The mean for child sex reflect the percentage of male students. CDSE, career decision-making self-efficacy.*

Prior to examining the whole model, the measurement model including all the exogenous, the mediator, and endogenous variables were included in a single model and correlated with each other. The factor loadings for the latent variable of autonomy were all significant at *p* < 0.001 (the loadings were 0.79 and 0.86 for emotional autonomy and behavioral autonomy, respectively) and the model fit the data well: χ^2^(7) = 4.26, *p* = 0.75, CFI = 1.00, RMSEA = 0.00 (90% CI [0.00, 0.03]), SRMR = 0.005, indicating an adequate measurement model.

**FIGURE 3:**
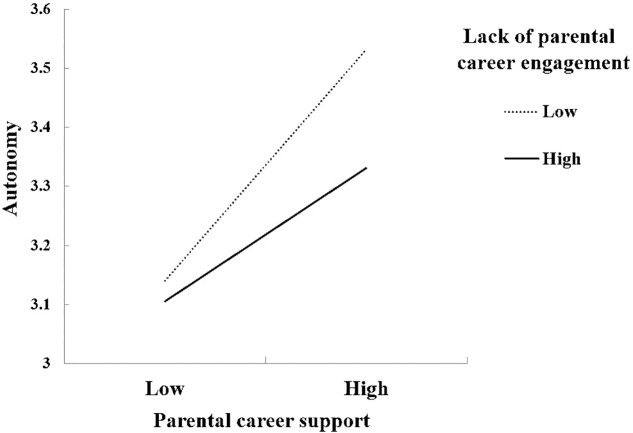
Parental career support interacted with lack of parental career engagement in the prediction of adolescents’ autonomy.

The integrative model that examined associations among three career-related parenting practices, autonomy, and CDSE, fit the data well (Figure 2): χ^2^(22) = 26.91, *p* = 0.21, CFI = 0.99, RMSEA = 0.02 with 90% CI [0.00, 0.04], SRMR = 0.021. The three-way interaction among parental career support, interference, and lack of engagement was not related to adolescents’ CDSE or autonomy. Two significant two-way interactions emerged. Parental career support interacted with parental lack of engagement when predicting adolescents’ autonomy (β = -0.22, *p* = 0.003; Figure 3). Specifically, the positive association between parental career support and adolescents’ autonomy was stronger among adolescents who reported low levels of parental lack of career engagement (-1 *SD*, β = 0.24, *p* < 0.001) than those who reported high levels of parental lack of career engagement (+1 *SD*, β = 0.14, *p* < 0.001).

**FIGURE 4:**
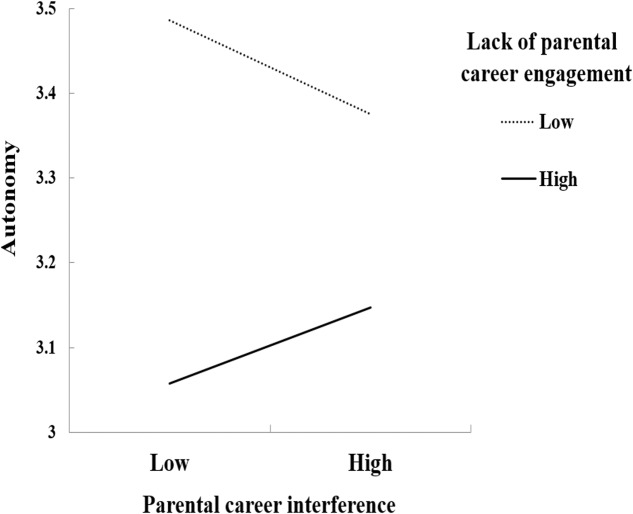
Parental career interference interacted with lack of parental career engagement in the prediction of adolescents’ autonomy.

Moreover, parental career interference interacted with lack of parental career engagement in the prediction of adolescents’ autonomy (β = 0.18, *p* = 0.05; Figure 4). Specifically, parental career interference was associated negatively with adolescents’ autonomy when lack of parental career engagement was low (-1 *SD*, β = -0.06, *p* = 0.001) but not when lack of parental career engagement was high (+1 *SD*, β = 0.04, *p* = 0.02).

Overall, two moderated mediating effects emerged (Table 2). Parental career support interacted with parental lack of engagement in relation to adolescents’ CDSE via autonomy: β = -0.13, 95% CI [-0.21, -0.04]. Specifically, the indirect effect for parental career support – autonomy – CDSE was larger when parental lack of engagement was low (i.e., parents displayed high levels of career engagement) (-1 *SD*, β = 0.20, 95% CI [0.13, 0.28]) than that when parental lack of engagement was high (i.e., parents displayed low levels of career engagement) (+1 *SD*, β = 0.12, 95% CI [0.08, 0.16]). Parental career interference also interacted with parental lack of engagement in relation to adolescents’ CDSE via autonomy: β = 0.11, 95% CI [0.03, 0.22]. Specifically, the indirect effect for parental career interference – autonomy – CDSE was negative when parental lack of engagement was low (-1 *SD*, β = -0.07, 95% CI [-0.10, -0.02]) than when parental lack of engagement was high (+1 *SD*, β = 0.04, 95% CI [0.00, 0.07]).

**Table 2:** Conditional indirect effects of different career-specific parental practices on CDSE through autonomy, at varying levels of lack of parent career engagement.

Variables	β	*SE*	95% CI
PS	**0.24**	**0.04**	**0.16, 0.32**
PI	-0.004	0.04	-0.08, 0.06
LP	**-0.02**	**0.05**	-0.34, -0.12
PS × PI	0.06	0.05	-0.03, 0.17
PS × LP	**-0.13**	**0.04**	**-0.21, -0.04**
PI × LP	**0.11**	**0.05**	**0.03, 0.22**
PS × PI × LP	0.02	0.06	-0.11, 0.12

	**Conditional indirect effects of PS on CDSE at specific values of LP**		

**Value of LP**	**Indirect effect**	***SE***	**95% CI**

-1 *SD* (-0.71)	**0.20**	**0.04**	**0.13, 0.28**
*M* (0.03)	0.16	0.02	0.12, 0.21
+1 *SD* (0.77)	**0.12**	**0.02**	**0.08, 0.16**

	**Conditional indirect effect of PI on CDSE at specific values of LP**		

**Value of LP**	**Indirect effect**	***SE***	**95% CI**

-1 *SD* (-0.71)	**-0.07**	**0.02**	**-0.10, -0.02**
*M* (0.03)	-0.01	0.02	-0.04, 0.02
+1 *SD* (0.77)	**0.04**	**0.02**	**0.00, 0.07**


*All parameter estimates and significance tests are based on 2,000 bootstrapped samples. SE, standard error. Significant effects are determined by both 95% CI that does not include zero and ps < 0.05, which are in bold typeface for emphasis. PS, parental career support; PI, parental career interference; LP, lack of parental career engagement; CDSE, career decision-making self-efficacy.*

## Discussion

Based on data from a large sample of Chinese adolescents, this study is among the initial efforts in examining the understudied unique and interactive associations between career-specific parenting practices and adolescents’ CDSE as well as the mediating roles of autonomy in explaining such associations. Identifying antecedents of adolescents’ CDSE is critical given that adolescence is an important developmental stage when they seek to learn about personal interests, values, and capabilities, ponder over the potential occupations that may suit their personal characteristics, develop stable identity, and prepare themselves for future career (Lee et al., 2016; Pellerone et al., 2017a).

### The Direct Association Between Career-Specific Parenting Practices and on Adolescents’ CDSE

Going beyond the general parenting behaviors and the sole focus on parental career support, this study extended the current research by revealing that adolescents who perceived higher career support from parents reported higher CDSE whereas adolescents perceived interference and lack of engagement from parents were not related directly with adolescents’ CDSE. The identified, unique association between parental career support and adolescents’ CDSE is consistent with the SCCT perspective and joints an emerging body of research highlighting the important role of parents’ career support in Chinese adolescents’ career development (Lent et al., 1994; Tracey et al., 2006; Keller and Whiston, 2008; Garcia et al., 2012; Zhang et al., 2015). On one hand, parents’ career instrumental support in the forms of encouragement, instrumental assistance, and modeling desired behaviors may provide necessary resources to enable career exploration and thus may lead to enhanced confidence and motivation to pursue their career goals (Kanten et al., 2016).

On the other hand, parents’ various forms of support also may create an atmosphere that encourages adolescents’ active exploration of their selves and environment and also set a stable basis for them to cope with career challenges and to conceive their future (Marcionetti and Rossier, 2017). Taken together, both parents’ instrumental and emotional support represent parents’ active involvement in adolescents’ career development and ultimately may promote adolescents’ CDSE. Future research is needed to differentiate between parents’ instrumental and emotional support in relation to adolescents CDSE, which holds critical implications for the development of targeted intervention programs.

### The Interactive Effects of Career-Specific Parenting Practices on Adolescents’ CDSE Through Autonomy

This study further advances the literature by examining the linking mechanism that underlies associations between career-specific parenting practices and adolescents’ CDSE. Although a self-determination perspective highlights the critical role of autonomy in fostering individuals’ development, to our knowledge, this study is one of the first efforts in revealing that autonomy served as the linking mechanism that explained the direct and interactive effects of career-specific parenting practices on adolescents’ CDSE.

Specifically, the lack of parental career engagement conditioned the association between parental career support and adolescents’ CDSE as well as the negative association between parental career interference and adolescents’ autonomy. The results indicated that whether parents’ engagement in their children’s career development (i.e., lower levels of lack of parental career engagement) foster either higher or lower CDSE via autonomy is contingent upon whether parents provide actual guidance and/or warmth (i.e., higher levels of parental career support) or over-control their children’s career direction or actions (i.e., higher levels of parental career interference).

These interactive effects are consistent with results of prior studies (Dietrich and Kracke, 2009; Guan et al., 2015). Specifically, the conveyance of competence and confidence in career exploration and making career decisions (i.e., CDSE) based on parents’ career guidance and warm support tends to be strengthened by parents’ active engagement (Lent et al., 1994, 2017). These findings also were consistent with the self-determination perspective such that parents’ high levels of career support (e.g., warmth, encouragement and provision of necessary career planning or exploration resources) coupled with parents’ active engagement may provide opportunities for adolescents to explore valuable information and experiences and thus to gain insights for future career via enhanced sense of autonomy (Deci and Ryan, 1985; Guay et al., 2003; Dietrich and Salmela-Aro, 2013). In contrast, when parents approach their adolescents’ career development with controlling behaviors (i.e., parental career interference), or without sufficient support may perceive a high level of difficulty in coping with and overcoming the challenges in their career development due to compromised intrinsic motivation associated with autonomy (Amarnani et al., 2018). These findings contributes to the literature by revealing *‘how’* the interplay of different career-specific parenting practices relate to adolescents’ CDSE (Deci and Ryan, 1985; Lent et al., 1994).

Furthermore, the amplifying effect of engagement in facilitating adolescents’ CDSE via providing career support also is consistent with a self-verification theory such that the positive social feedback engendered by parental career support is likely to be facilitated by heightened parental engagement (Swann et al., 1992). In contrast, in the context of parents’ ignorance or incapability to provide guidance for adolescents’ career development, adolescents are prone to deviate from career exploration and actions and thus may lead to adolescents’ compromised autonomy and ultimately undermined confidence in making career decisions (Amarnani et al., 2018). However, parents’ active career engagement coupled with intrusive and controlling career involvement (i.e., parents’ imposing their own thoughts on their children’s career directions and explorations) tend to undermine adolescents’ autonomy given parental career interference imply to adolescents that they are not able to effectively engage in career exploration, establish career direction and goal, and/or make adaptive career decisions (Dietrich and Kracke, 2009; Guan et al., 2015).

### Implications for Practice

As there is not yet national, official career guidance programs for high school students in China, Chinese parents’ career-specific parenting behaviors may play a significant role in facilitating Chinese adolescents’ career development. The examination of the relationships between different career-specific parenting behaviors and Chinese high school students’ CDSE may have important implications for Chinese high school’s career guidance practice. First, parenting-based interventions to promote adolescents’ CDSE is justified in the Chinese context. Specifically, school psychologists may find it useful to tailor interventions to increase students’ CDSE through targeting their parents’ career-specific parenting behaviors and enhancing their autonomy. Second, although more and more Chinese high schools begin to develop career guidance programs in recent years (Hu et al., 2015), there are only a limited number of counselors in Chinese high schools. As such, it is important to identify students who need more intensive career counseling interventions (Hu et al., 2015). The present study provides potential directions to identify potential clients, like those students who perceived their parents displaying high levels of career-related engagement and over-controlling parenting practices. Moreover, the present study highlights the importance of strengthening school-family partnerships, which may provide students with parental career involvement and autonomous support for their adolescent kids, which may ultimately facilitate students’ CDSE (Watkinson and Hersi, 2014).

### Limitations and Future Directions

The present study should be considered in light of its limitations. First, as adolescents reported career-specific parenting practices and CDSE on their own, some of the identified associations may be inflated. Future studies are warranted to employ multi-informant and multi-method designs to minimize shared informant and method bias. Second, given the widespread grandparents’ heavy involvement in Chinese adolescents’ daily lives and potential different parenting practices executed by grandparents from parents (Chen et al., 2011), it is not clear how career-specific parenting practices and adolescents’ CDSE may vary between adolescents raised mainly by grandparents and those by parents. Finally, given the cross-sectional nature of the data used, the presents study cannot address the temporal dynamics of the examined associations. Future research with longitudinal designs is warranted.

## Ethics Statement

This study was carried out in accordance with the recommendations of Ethics Review Committee at the School of Psychology, Beijing Normal University with written informed consent from all the participants. All the adolescents and their parents gave written informed consent in accordance with the Declaration of Helsinki. The protocol was approved by the Ethics Review Committee at the School of Psychology, BNU.

## Author Contributions

YZ, NZ, HC, YL, QW, and XF designed the study and drafted the manuscript. YZ, RS, PL, QX, and RN performed the research. YZ and NZ analyzed the data and revised the manuscript. SY, JL, LD, and RS re-analyzed the data and revised the manuscript. All authors approved the final version of manuscript for submission.

## Conflict of Interest Statement

The authors declare that the research was conducted in the absence of any commercial or financial relationships that could be construed as a potential conflict of interest.
